# Fanconi Syndrome Leading to Hypophosphatemic Osteomalacia Related to Tenofovir Use

**DOI:** 10.3390/idr13020044

**Published:** 2021-05-24

**Authors:** Mana Rao, Liam Dadey, Thomas Glowa, Peter Veldkamp

**Affiliations:** 1 Essen Medical Associates Bronx, New York, NY 10453, USA; 2 Parkview Health, Fort Wayne, IN 46805, USA; Liam.Dadey@parkview.com; 3 Division of Infectious Diseases, Department of Medicine, University of Pittsburgh Medical Center, Pittsburgh, PA 15213, USA; glowatw@upmc.edu (T.G.); pjv4@pitt.edu (P.V.)

**Keywords:** tenofovir, Fanconi syndrome, hypophosphatemic osteomalacia, HIV, adverse event

## Abstract

Tenofovir disoproxil fumarate (TDF) is used worldwide to treat and prevent Human Immunodeficiency Virus (HIV) infection. Fanconi syndrome is a complication of TDF use and is characterized by inadequate reabsorption of glucose, phosphate and protein in the proximal tubule of the kidney which may eventually lead to osteomalacia manifested by symptoms of pain, muscular weakness and difficulty ambulating. We present a patient with severe osteomalacia due to progressive and unrecognized Fanconi’s syndrome, who responded rapidly to TDF withdrawal, oral phosphate repletion and calcitriol. With the widespread use of TDF-containing antiviral regimens, it is critically important that physicians adhere to screening recommendations to detect early Fanconi syndrome, and recognize symptoms of osteomalacia as a serious complication.

## 1. Introduction

The global prevalence of Human Immunodeficiency Virus (HIV) in 2017 was estimated to be 36.9 million by UNAIDS, of whom 21.7 million were reported to have been on antiretroviral therapy (ART) [1]. HIV related mortality has been on a steady decline since 2006, and this is due to the availability and use of combination ART. Several fixed-dose combination ART medications, namely Atripla, Complera, Stribild and Truvada have a tenofovir disoproxil fumarate (TDF) backbone.

World Health Organization (WHO) guidelines on HIV recommend the use of TDF or tenofovir alafenamide (TAF) based ART as an initial regimen for most people with HIV [2]. While the pro-drug TAF has few or no renal toxicity compared to TDF, due to decreased serum exposure [3], its high cost precludes widespread substitution for TDF. A large number of the HIV population worldwide are being exposed to TDF-based ART regimens. Furthermore, TDF is used to treat hepatitis B virus (HBV), and Truvada (TDF with emtricitabine) remains a frontline recommendation for medical HIV prevention. Thus, a large number of people are exposed to TDF.

Though TDF provides compelling benefits in HIV treatment, it has been known to cause renal dysfunction leading to significant morbidity. The incidence of Fanconi syndrome in patients using TDF has been reported to be 5.50/1000 person years (PY) [4].

We describe a patient with severe osteomalacia and hypophosphatemia from Fanconi syndrome, who responded rapidly to TDF withdrawal, oral phosphate (PO_4_) repletion and calcitriol.

## 2. Case Presentation

A 54-year-old female with HIV virally suppressed on TDF, emtricitabine and efavirenz (Atripla) for the preceding 10 years, presented with progressive, intractable bilateral hip and lower extremity pain along with weakness in the lower extremities, progressive gait disturbance, and recurrent falls over 18 months. Her other medications included vitamin D 400 IU daily, calcium carbonate 600 mg daily, and nonsteroidal anti-inflammatory drugs (NSAIDs) which the patient reported were used sparingly for pain with incomplete relief. A serum creatinine done nine months prior was 1.2 mg/dl [estimated glomerular filtration rate (eGFR) 47 mL/min]. Mild qualitative proteinuria was present for two years prior to this presentation.

On exam, the patient was afebrile and had normal vital signs. Bilateral lower extremity strength was decreased and noted to be 4/5. A detailed musculoskeletal exam was limited by excruciating pain upon lower extremity range of movement. Upper extremity strength and mobility were intact. Laboratory studies revealed a normal blood count, and abnormal metabolic panel with hyperchloremia, acidosis, elevated serum creatinine and alkaline phosphatase. The serum phosphate level was decreased, the calcium level normal, and the Parathyroid hormone (PTH) level was elevated. Notably, Vitamin D levels were within reference ranges. Detailed laboratory values are listed in Table 1.

A spot urine test showed new onset glycosuria and phosphaturia, with a calculated fractional PO_4_ excretion of 62.3% (reference range <5%).

**Figure 1:**
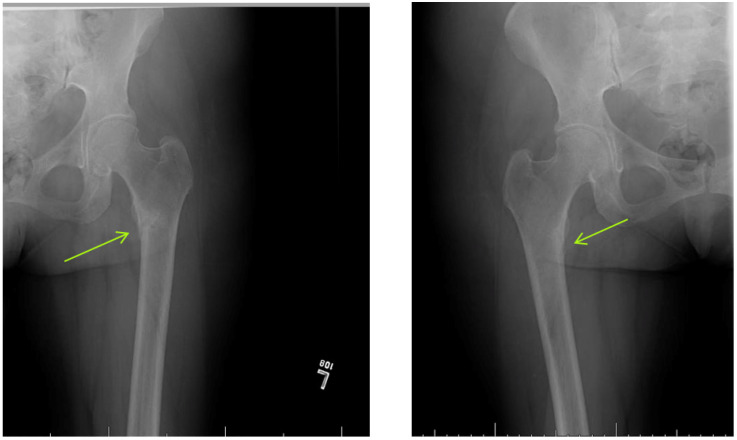
Hip/Femur X-Ray. Patchy regions of sclerosis in the medial subtrochanteric regions, Left (L) >Right (R) corresponding to uptake on bone scan concerning for subacute incomplete fractures. Consistent with Looser’s Zones.

**Figure 2:**
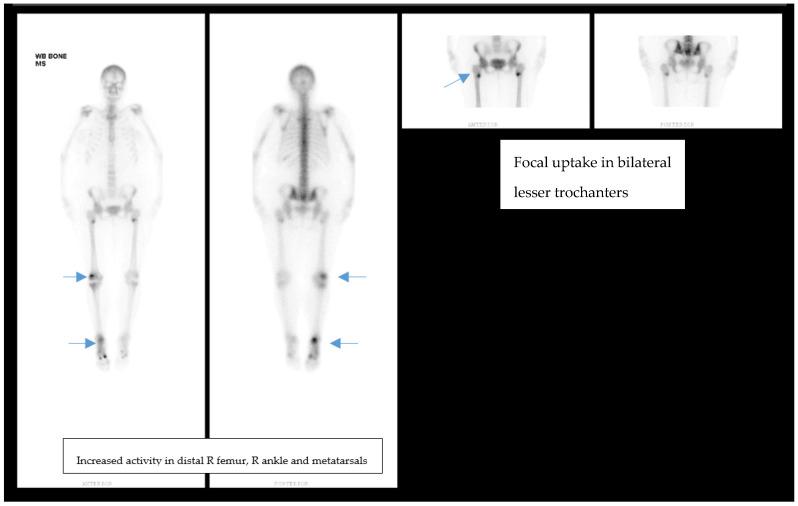
Bone scan of patient showing nonspecific focal uptake in bilateral lesser trochanters, read as compatible with chronic bone remodeling/increased bone turnover. Increased activity in distal R femur, R lateral tibial plateau. Increased activity in R ankle and 1st/3rd metatarsals. Radiotracer activity at L tenth rib.

Endocrinology was consulted for “unexplained hypophosphatemia” in the setting of bone pain. The patient’s laboratory studies were consistent with a hyperchloremic non-anion gap metabolic acidosis, consistent with proximal renal tubular acidosis (RTA) attributed to Fanconi syndrome. Skeletal X-ray studies revealed the presence of bilateral subtrochanteric Looser’s Zones with non-displaced proximal diaphyseal fractures of the first and third metatarsals of her right foot (Figure 1). A bone scan demonstrated increased uptake bilaterally in the lesser trochanter region of the proximal femur, distal right femur, right ankle, and the right first and third metatarsals (Figure 2).

Based on these findings, a diagnosis of TDF-related Fanconi syndrome with associated hypophosphatemia and osteomalacia was established. TDF was replaced by abacavir after human leukocyte antigen (HLA) testing was negative for the B5701 allele. Repletion with oral PO_4_ 250 mg three times daily, and calcitriol 0.25mcg daily was commenced. The patient experienced complete clinical, biochemical (Table 1) and radiographic resolution within 14 weeks of follow up, allowing for discontinuation of oral phosphate and calcitriol and initiation of maintenance cholecalciferol at 2000 IU/day. A DEXA scan performed following resolution of her symptoms demonstrated T scores at the forearm of −2.4, at the femoral neck of −0.9, and total hip of −0.3. There was no evidence of axial osteopenia (L1-L4 T score +2.1).

A written informed consent was obtained from the patient for publication of this case.

## 3. Discussion

TDF is a nucleoside reverse transcriptase inhibitor (NRTI) and is worldwide one of the most used components in HIV treatment and prevention as well as HBV suppressive therapy. The mechanism of action of tenofovir is selective inhibition of the viral reverse transcriptase enzyme, thereby preventing viral replication. It is excreted chiefly by the kidneys, both by glomerular filtration and tubular secretion. It is known to be a largely well tolerated drug. Nonetheless, it carries a risk of renal injury.

The spectrum of renal disease associated with TDF can vary from Fanconi syndrome to end stage kidney disease [5,6]. Clinically, one may note acute kidney injury (as in our case), or worsening chronic kidney disease. TDF induced renal disease may be associated with elevated baseline serum creatinine, older age, advanced HIV infection, low body weight, hepatitis C coinfection and concurrent use of other nephrotoxic drugs [7]. Boosted protease inhibitor (PI) use e.g., ritonavir along with TDF has also been shown to increase risk of renal injury due to a drug–drug interaction that may or may not be able to be avoided.

Fanconi syndrome is a disorder of inadequate reabsorption of phosphorous, glucose and amino acids at the level of the proximal convoluted tubule. It can be caused by genetic conditions (cystinosis, fructose intolerance, galactosemia and glycogen storage diseases), drugs (including TDF, adefovir, cisplatin, ifosfamide, deferasirox and valproic acid) [8,9,10] and heavy metals such as lead, mercury and cadmium.

The diagnosis of TDF-induced Fanconi syndrome is based on typical findings of glycosuria (with normal plasma glucose), proteinuria and phosphaturia (high fractional PO_4_ excretion) along with hypophosphatemia. Also notable is a non-anion gap hyperchloremic metabolic acidosis due to renal bicarbonate losses. Urine and blood tests to screen for these features facilitate early diagnosis of Fanconi syndrome. Notably, elevations in eGFR, which reflect glomerular and not tubular dysfunction, can be delayed by several years and therefore should not be relied upon for screening or diagnosis.

Osteomalacia is a bone disorder, characterized by decreased mineralization of newly formed osteoid at sites of bone turnover. It can occur due to disorders of calcium and/or phosphorus metabolism, or due to direct inhibition of bone mineralization. Osteomalacia may be asymptomatic or present with diffuse bone and joint pain, muscle weakness, and difficulty walking [11,12,13]. Other clinical features include bone tenderness and fractures.

Radiographically, pseudofractures, also known as Looser’s Zones or Milkman’s Fractures, are the most recognized feature of osteomalacia. This occurs secondary to impaired bone mineralization and increased PTH secretion. Lucent bands appear perpendicular to the periosteum and these usually develop in the outer border of the scapulae, ribs, pubic rami, femoral neck, and to a lesser extent in metacarpals and shafts of long bones. A bone biopsy is diagnostic, however, and is infrequently performed once clinical and laboratory diagnoses have been established corroborating a patient’s history and exam. Laboratory findings vary based on the etiology of osteomalacia. Commonly noted abnormalities are elevated alkaline phosphatase, reduced calcium and phosphorus, low urinary calcium, low 25-OH vitamin D, and elevated PTH [13,14].

Although TDF associated bone density loss mechanisms remain elusive, this has been proposed to be due to the fact that the TDF molecule is a phosphonate. It could influence osteoclast and osteoblast function, altering their gene expression and thereby their cellular function [15].

Osteomalacia and Fanconi syndrome in conjunction have been reported to be caused by adefovir in patients being treated for HBV [16,17,18,19].

Conjoined Fanconi syndrome and osteomalacia have also been reported to be caused by synovial sarcoma and giant cell tumors of bone [20].

The incidence and prevalence of TDF-related Fanconi syndrome with osteomalacia and hypophosphatemia are not well defined. Specifically, the prevalence of bone toxicity is not well established as only severe cases have been reported. Woodard et al. report a 0.12% incidence of hypophosphatemic osteomalacia among their patients [21].

Mateo et al. reported five cases of TDF induced hypophosphatemic osteomalacia wherein the patients had a HIV diagnosis established at least 18 years prior, were symptomatic for >6 months, and three out of five cases had a low 25 (OH) Vitamin D level.

Three patients were treated only with calcium and vitamin D, while the other two needed phosphate supplementation. In all cases, bone pain disappeared after TDF cessation [22].

Parsonage et al. have reported two cases of hypophosphatemic osteomalacia with myopathy among HIV patients on TDF. Withdrawal of TDF and mineral supplementation was their treatment of choice, with complete clinical and biochemical resolution in both cases [23].

Clinical and laboratory findings of hypophosphatemic osteomalacia with Fanconi syndrome due to TDF are identical to those found in the individual entities listed above. Early recognition of this syndrome is vital and feasible. Removal of the inciting agent is critical for clinical resolution, which may occur well before normalization of biochemical or radiographic abnormalities.

Current guidelines recommend screening all patients on TDF or TAF biannually for measurements of renal function and urinalysis [24]. While bone and renal toxicities are felt to be much lesser with TAF compared to TDF, long term data are needed for head to head comparison. As previously stated, the use of TAF is limited still due to high cost and limited availability in the global market.

## 4. Conclusions

The diagnosis of TDF-induced Fanconi syndrome is based on the findings of glycosuria, proteinuria, and phosphaturia (high fractional PO_4_ excretion), as well as hypophosphatemia. Biannual urine and blood tests to screen for these features facilitate early diagnosis of Fanconi syndrome. Notably, decrease in eGFR can be delayed by several years in Fanconi syndrome. Furthermore, recognition of osteomalacia depends on radiographic screening for pseudofractures in the context of proximal (Type 2) RTA and typical musculoskeletal symptoms. Fanconi syndrome and hypophosphatemic osteomalacia can occur simultaneously in the presence of TDF as the inciting agent. These clinical entities may also occur independently as a result of long term TDF use. Removal of TDF is critical for clinical resolution, which may occur well before normalization of biochemical or radiographic abnormalities.

## Figures and Tables

**Table 1:** Laboratory values at presentation and patient on follow up.

	At Presentation	2–3 Months after Hospitalization	Reference Range
Laboratory Testing			
Calcium (mg/dl)	8.6	9.8	8.4–10.2
Phosphate (mg/dl)	1.7	4.5	2.5–4.6
25-OH Vitamin D (ng/mL)	33	47 (at 4 months)	25–100 “adequate” >30
1,25(OH)2 vitamin D (pg/mL)	48		18–72
Chloride (meq/L)	113	108	98–107
Bicarbonate (meq/L)	20	22	21–31
Alkaline Phosphatase (IU/dl)	258	280	38–126
Creatinine (mg/dl) (baseline 1.2)	1.7	1.3	
eGFR (ml/min)	31	43	
PTH (pg/mL)	80	58	10–65
Fractional PO4 excretion, urine (%)	62.3		<5
UA	+250 glucose, +300 protein	+100 protein, +100 glucose	

## Data Availability

All relevant information has been presented in the case report.
